# Supplementary material for: Charge-separation driven mechanism via acylium ion intermediate migration during catalytic carbonylation in mordenite zeolite
Source: Nat Commun. 2022 Nov 19;13:7106. doi: 10.1038/s41467-022-34708-5 (PMC9675746; doi:10.1038/s41467-022-34708-5)
Supplement: Supplementary file 1 — Supplementary Information [file 41467_2022_34708_MOESM1_ESM.pdf]

Supporting Information for

Charge-separation Driven Mechanism via Acylium ion Intermediate Migration during Catalytic Carbonylation in Mordenite Zeolite

Wei Chen,[1] Karolina A. Tarach,[2] Xianfeng Yi,[1] Zhiqiang Liu,[1] Xiaomin Tang,[1] Kinga Góra-Marek,[2, *] and Anmin Zheng,[1, 3, *]

[1]*State Key Laboratory of Magnetic Resonance and Atomic and Molecular Physics, National Center for Magnetic Resonance in Wuhan, Innovation Academy for Precision Measurement Science and Technology, Chinese Academy of Sciences, Wuhan 430071, P. R. China.*

[2]*Faculty of Chemistry, Jagiellonian University in Krakow, Gronostajowa 2, 30-387 Krakow, Poland.*

[3]*University of Chinese Academy of Sciences, Beijing 100049, P. R. China*

Corresponding authors:

Anmin Zheng, *E-mail:* zhenganm@wipm.ac.cn

Kinga Góra-Marek, *E-mail:* kinga.gora-marek@uj.edu.pl

This file includes:

Supplementary Methods

Supplementary Discussion

Supplementary Figures 1-26

Supplementary Tables 1-2

Supplementary References 1-13

Supplementary Methods

Enhanced sampling methods Herein, the different CVs between MOR-8MR and MOR-12MR were chosen to well describe the MA formation in different confined spaces. In MOR-8MR, methanol cannot well induce the C-O bond rupture of surface acetyl due to the limitation of the narrow space of side pocket and 8MR channel, therefore, the bias potential was performed on the coordination number of C-O bond in surface acetyl for the formation of acylium ion firstly, and then acylium ion with the high mobility can bond to methanol for the formation of MA. But in MOR-12MR, the inducing effect of methanol or DME to surface acetyl can play their full role to form MA, and the bias potential to the coordination number of C-O bond in surface acetyl will lead to the formation of acylium ion and further deprotonate to ketene as the side reaction. In summary, the different chances to CVs as reaction coordinates in MOR-8MR and MOR-12MR is exactly to better describe the reaction after many attempts, and our current setting to CVs in all models fulfill the requirements to reaction coordinates proposed by Peters.[1]

Error bars of free energy in MTD and US simulations In order to determine the error bars, the simulation was continued such that a further 1000 hills were added to the system after convergence. The error bar with respect to the transition state was then calculated as the average between the energy barrier at the moment of convergence and the energy barrier one these 200 additional energy hills had been included (Supplementary Fig. 23).[2] The error bars of free energy profile in US simulation were directly generated with free energy profile by weighted histogram analysis method (Supplementary Fig. 24).[3]

Energy decomposition analysis Energy decomposition analysis (EDA) was used to quantificationally describe the host-guest interaction of TS3 (Fig. 1d) in different channels of MOR zeolite. Detailed and precise analyses of host-guest interactions may be accomplished based on the ETS-NOCV EDA approach,[4] which combined the extended transition state (ETS) method with the natural orbital for chemical valence (NOCV) theory by means of the Amsterdam density functional (ADF) program.[5] The PBE-D3 functional was chosen for the DFT calculations and EDA to warrant the uniformity between dynamic and static calculations. By invoking the frozen core approximation, a double-zeta Slater type orbital (STO) basis set containing polarization functions (namely, DZP) was adopted for all elements to describe interactions between intermediates and the zeolite framework.[6] Auxiliary STO functions, centered on all nuclei, were used to fit the electron density and to obtain accurate Coulomb potentials in each SCF cycle. For the ETS-NOCV EDA scheme, the interaction energy (ΔE_{total}) between the fragments may further be divided into four components:

$$\Delta E_{total} = \Delta E_{Pauli} + \Delta E_{elec} + \Delta E_{orb} + \Delta E_{disper}. \quad (1)$$

Where the four energy terms respectively account for the Pauli repulsive interaction among occupied orbitals on the fragments (ΔE_{Pauli}), the classical electrostatic interaction between two fragments (ΔE_{elec}), electron distribution of the constituent molecules (ΔE_{orb}), the dispersion interaction (ΔE_{disper}) due to the use of dispersion corrected PBE-D3 functional.

Reduced density gradient analysis To visualize pore confinement between the adsorbed Hammett indicators and the zeolite channels, the noncovalent interaction index approach, developed by Yang *et al.*,[7] was adopted as well. In this approach, the reduced density gradient (RDG), defined as $s = (1/(2(3\pi^2)^{1/3}))((|\Delta\rho(r)|)/(\rho(r)^{4/3}))$, together with the electron density ρ, was used to distinguish the covalent and noncovalent interactions. The noncovalent interactions were located in the regions with low density and low RDG. The sign of the second largest eigenvalue (λ_2) of the electron density Hessian can be used to distinguish bonded ($\lambda_2 < 0$) from nonbonded ($\lambda_2 > 0$) interactions. The analysis of the sign of λ_2 can help to discern different types of noncovalent interactions: $\text{sign}(\lambda_2)\rho < 0$, H-bonding interactions (blue); $\text{sign}(\lambda_2)\rho \approx 0$, weak van der Waals (vdW) interactions (green) and $\text{sign}(\lambda_2)\rho > 0$, strong repulsive interactions (red). To exhibit the intermolecular noncovalent interactions between the adsorbed Hammett indicators and the zeolite framework more obviously, the intramolecular interactions were eliminated for the calculated RDG function. The functions RDG and $\text{sign}(\lambda_2)\rho$ were calculated using Multiwfn software[8] and visualized by VMD software.[9]

Sampling analysis of MTD simulations The sufficiency of bias sampling to product has been confirmed by the converged free energy profiles in Supplementary Fig. 22. To evaluate the sufficiency of bias sampling to reactant and transition states in MTD simulation, the probability distribution functions of approximation to the CV1 in the MTD simulation of surface acetyl + MeOH to MA + BAS in MOR-8MR were used to evaluate the probability density of the reactant configurations, and the high probability of the reference reactant states (CV1 = -1.03) indicates the sufficient sampling to reactants as displayed in Supplementary Fig. 25. Moreover, the radial distribution function for the atoms pair of C_{acetyl} and O_{Zeo} during the MTD simulation of surface acetyl + MeOH to MA in MOR-8MR was used to evaluate the sufficiency of sampling to transition state as displayed in Supplementary Fig. 26, and the efficient and reasonable distribution of $d(C_{acetyl}\text{-}O_{Zeo})$ at the transitional range of bond rupture (1.70 Å ~ 2.00 Å) indicate the enough sampling to transition state. In summary, the sufficient sampling to both reactant and transition state guarantee the accuracy of free energy profiles.

Chemical shift Calculation The chemical shift of acylium ion in side pocket of mordenite zeolite was calculated by B97-2 functional[11] in Gaussian 09 program,[10] pcSseg-1 basis set[12] was used to describe all elements in the cluster model. This method shows the high accuracy to predict the ^{13}C chemical shift.[13]

Supplementary Discussion

More details of acylium ion diffusion Unlike the acylium ion migration without additional BAS in the 12MR channel (Fig. 4), the migrated acylium ion will transfer to surface acetyl in the presence of additional BAS in the 12MR channel, and the formed surface acetyl will lead to the weak Al-O(H) bond as displayed in Supplementary Fig. 16a. Moreover, the introduction of DME/MeOH in the 12MR channel will not lead to spontaneous reaction between acetyl species and DME/MeOH (Supplementary Fig. 16b and S16c), and the DME/MeOH will be firstly protonated by additional BAS and migrated acylium ion will directly transfer to surface acetyl via the C-O bond coupling between positive charged acylium ion and negative charged AlO_4^-. In this context, the acylium ion completely transfer to the surface acetyl in the 12MR channel and the formation of MA will overcome the extra free barrier as illustrated in Fig. 2a and 2b. Overall, the existence of BAS in the 12MR channel is detrimental to the migration of acylium ion and the spontaneous MA formation.

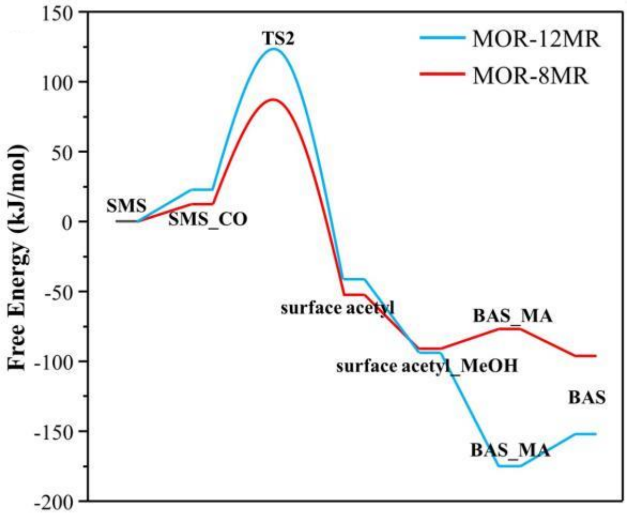

Supplementary Fig. 1. Free energy profile of MeOH carbonylation in MOR-12MR and MOR-8MR at 473 K.

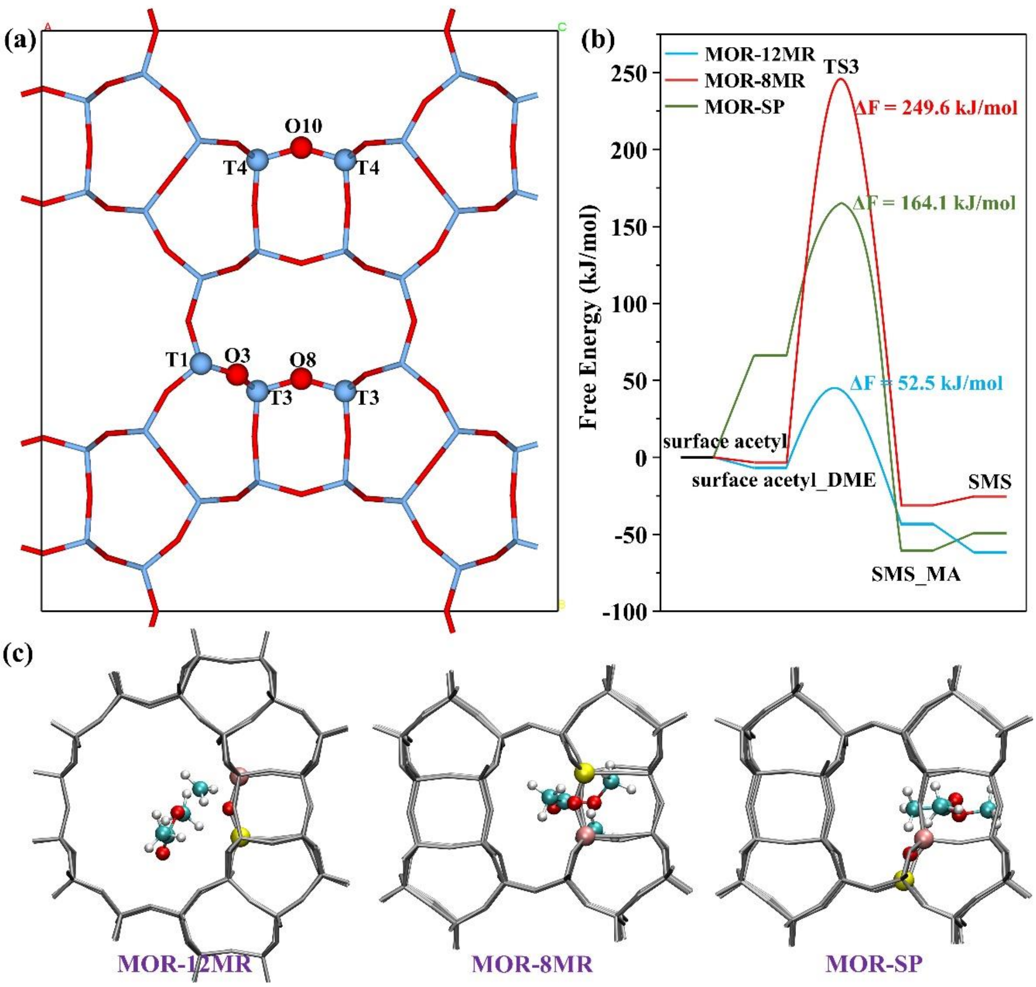

Supplementary Fig. 2. MA formation in different channels of mordenites. (a) T sites and oxygen of mordenite zeolite to bind surface acetyl in MOR-12MR (T4-O10-T4), MOR-8MR (T3-O8-T3), and MOR-SP (T3-O3-T1), (b) free energy profile of MA formation via surface acetyl and DME in MOR-12MR, MOR-8MR, and MOR-SP at 473 K, (c) geometrical structure of TS3b in MOR-12MR, MOR-8MR, and MOR-SP.

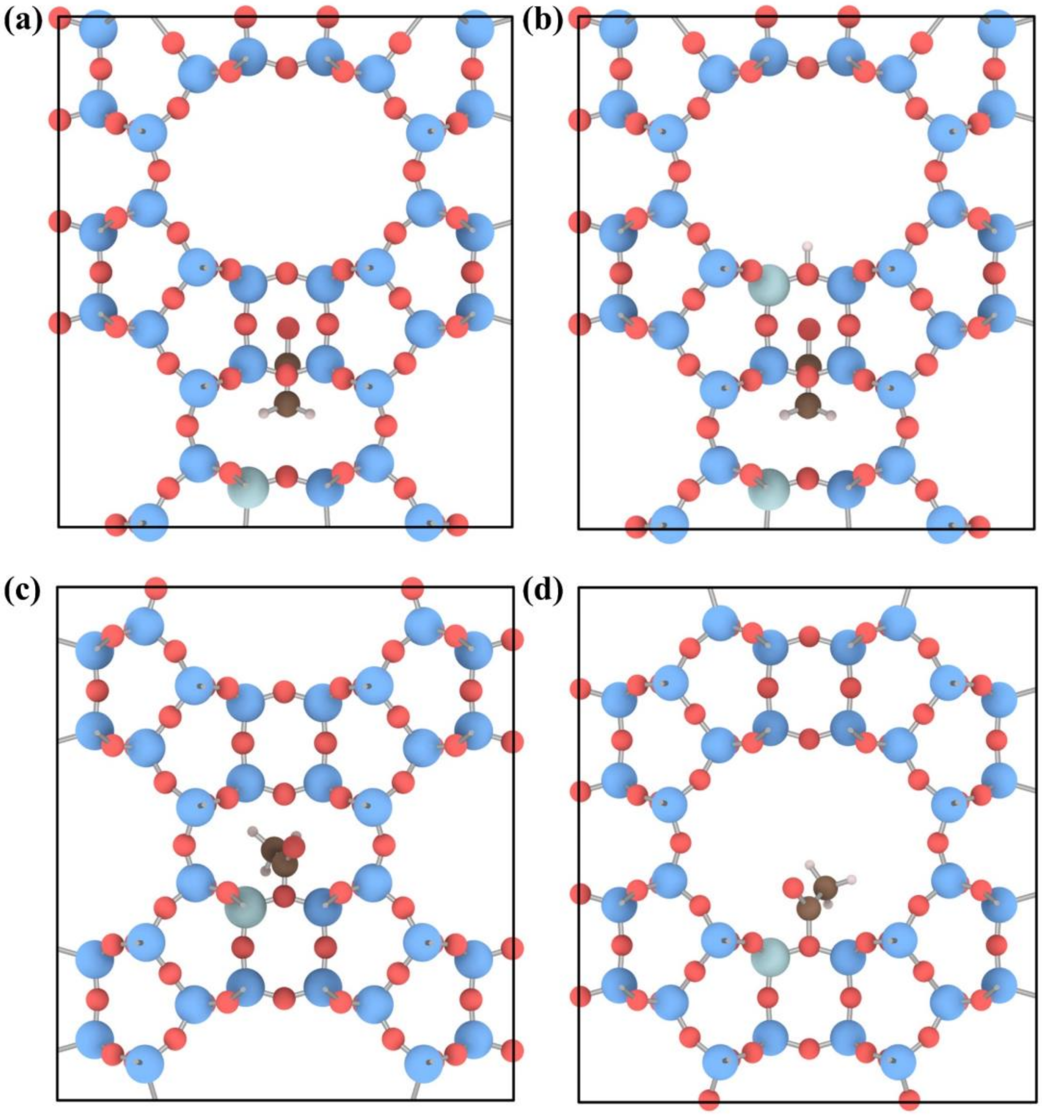

Supplementary Fig. 3. Periodic structure models of mordenite zeolite in this work. (a) acylium ion in side pocket with Si-O^{-}-Al (b) acylium ion in side pocket with Si-O^{-}-Al and additional BAS in 12MR channel, (c) surface acetyl in 8MR channel of mordenite, (d) surface acetyl in 12MR channel of mordenite.

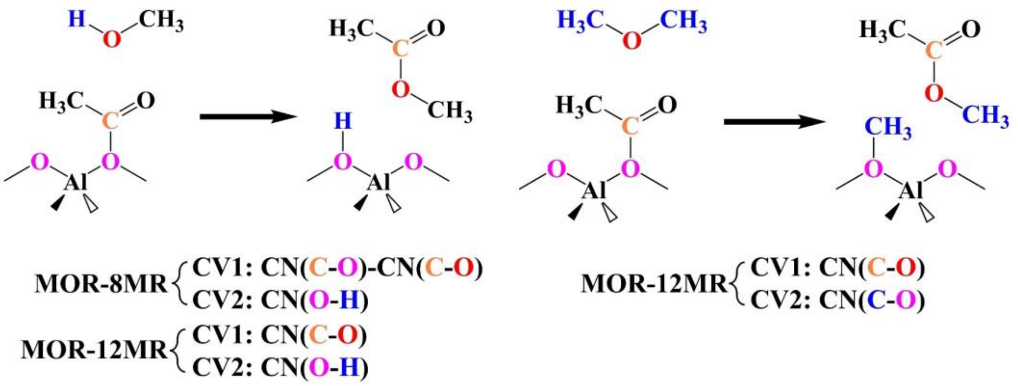

Supplementary Fig. 4. Collective variables (CV) to accelerate the MA formation in mordenite zeolites *via* surface acetyl and MeOH (or DME) in AIMD-MTD simulation. For the methanol + surface acetyl to MA in MOR-8MR, the reaction process takes place by backside attack of MeOH in the side pocket on the surface acetyl, here, the reaction is hampered by proceeding through direct frontal attack due to space constraints in the side pocket facing the surface acetyl.

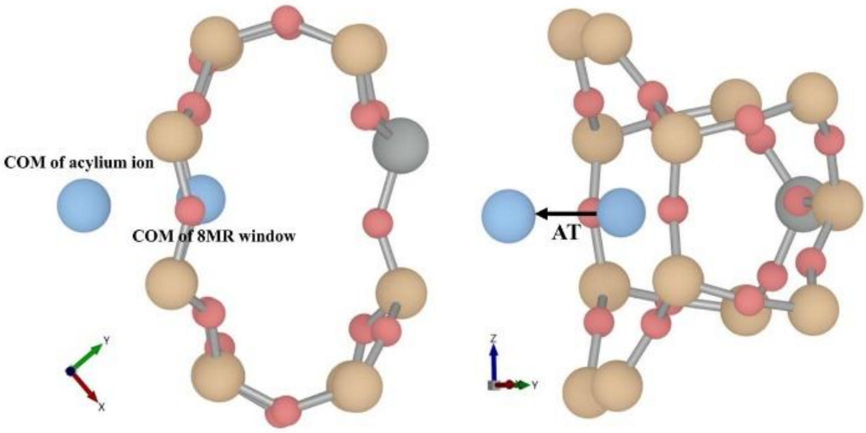

Supplementary Fig. 5. The mobility of acylium ion from 8MR channel to 12MR channel was controlled by the distance between two centers of mass (COM) in AIMD-US simulation. one is the COM of 8MR window face to the Si-O⁻-Al, the other is the COM of acylium ion. AT is this distance in every US-AIMD simulations. AT is set to the integer from 2Å to 9Å in ten individual US-AIMD simulations.

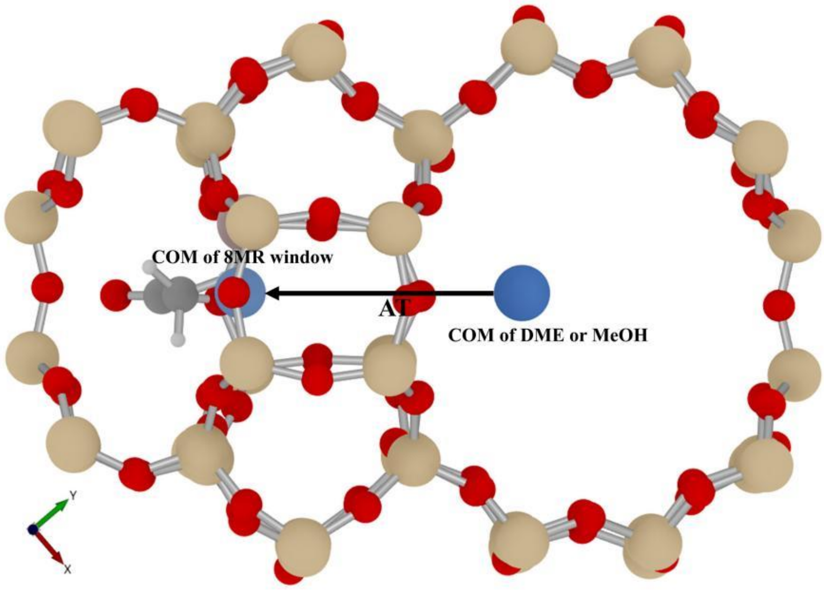

Supplementary Fig. 6. The mobility of DME or MeOH from 12MR channel to 8MR channel in the presence of surface acetyl was controlled by the distance (AT) between two centers of mass (COM) in AIMD-US simulation. one is the COM of 8MR window including the Si-O^{-}-Al, the other is the COM of DME or MeOH. AT is set to the integer from 2 Å to 9 Å in eight individual US-AIMD simulations.

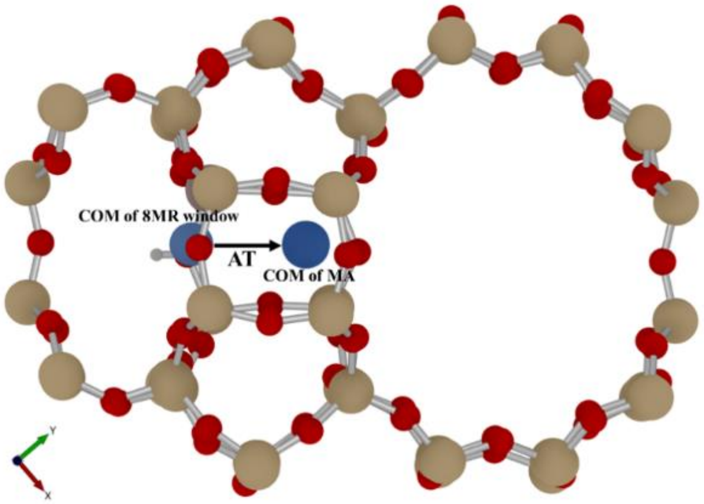

Supplementary Fig. 7. the mobility of MA from 8MR channel to 12MR channel in the presence of BAS was controlled by the distance (AT) between two centers of mass (COM) in AIMD-US simulations, one is the COM of 8MR window including the BAS, the other is the COM of MA. AT is set to the integer from 0 Å to 8 Å in nine individual US-AIMD simulations.

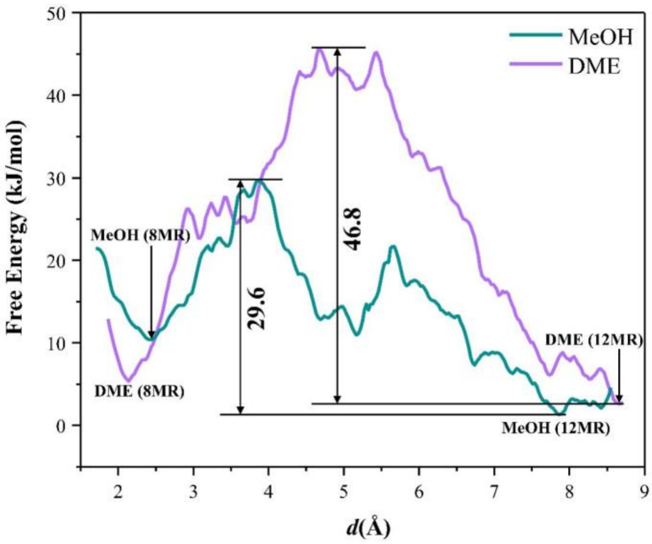

Supplementary Fig. 8. Free energy profile of MeOH and DME diffusion from 12MR channel to 8MR channel in the presence of one surface acetyl in 8MR channel of MOR.

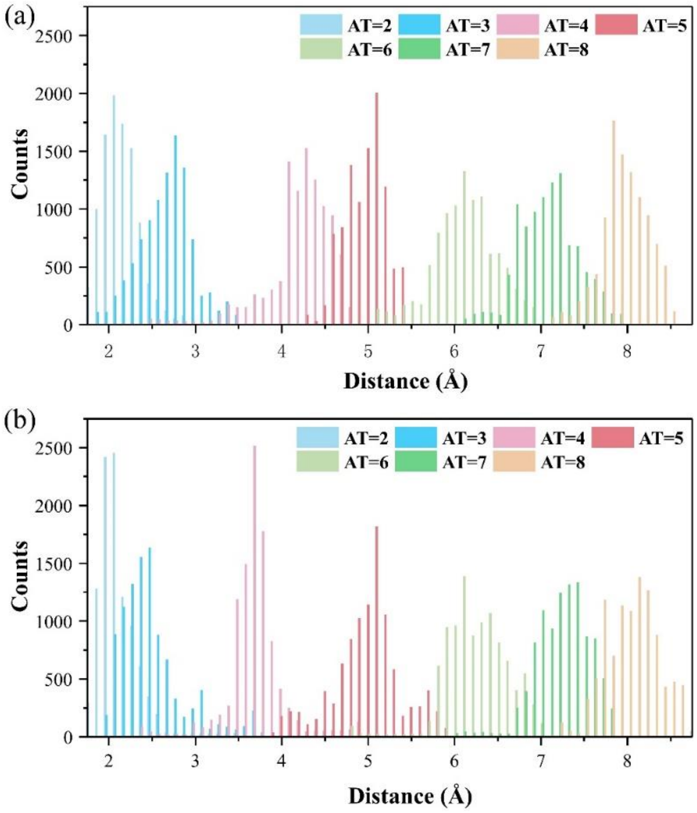

Supplementary Fig. 9. The distribution of distance in every independent umbrella sampling with different AT for (a) DME or (b) MeOH transport from 12MR channel to 8MR channel in the presence of surface acetyl in 8MR channel.

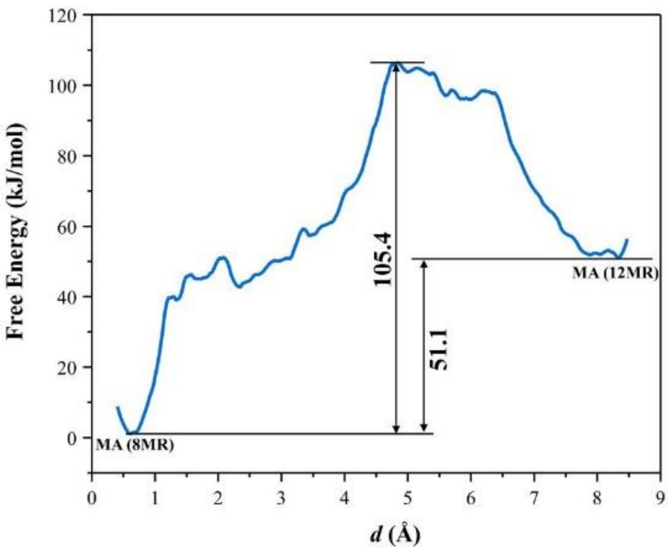

Supplementary Fig. 10. Free energy profile of MA diffusion from 8MR channel to 12MR channel in MOR in the presence of SMS in 8MR channel.

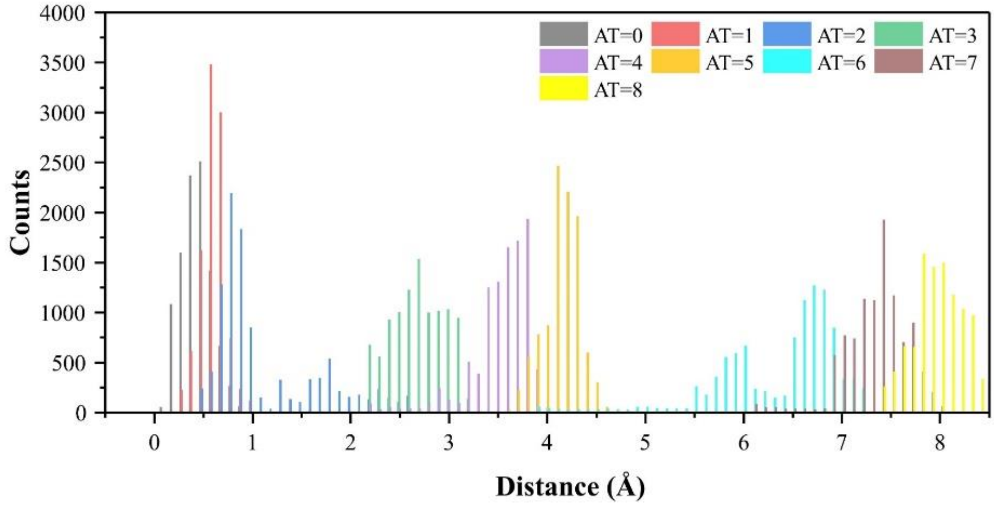

Supplementary Fig. 11. The distribution of distance in every independent umbrella sampling with different AT for MA transport from 8MR channel to 12MR channel in the presence of BAS in 8MR channel.

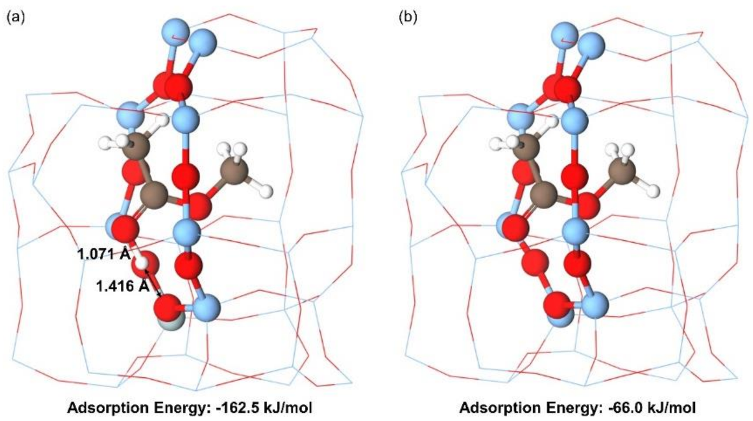

Supplementary Fig. 12. Adsorption energy and structure of MA in MOR-8MR (a) with and (b) without BAS.

Supplementary Table 1. Adsorption free energy (kJ/mol) of DME and MeOH in surface acetyl and acylium ion containing MOR-12MR and MOR-8MR at 473 K.

	DME	MeOH
MOR-12MR_surface acetyl	63.3	45.6
MOR-8MR_surface acetyl	45.3	55.2
MOR-8MR_acylium ion[a]	57.0	56.3

[a]DME and MeOH was adsorbed in 12MR channel of mordenite.

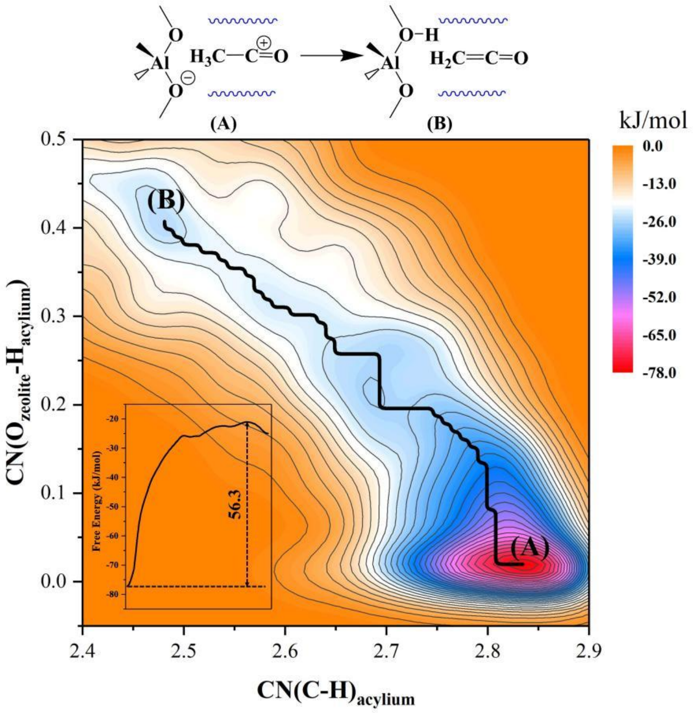

Supplementary Fig. 13. Free energy profile of acylium ion which deprotonate to ketene in 8MR channel of MOR. (A) acylium ion, (B) ketene.

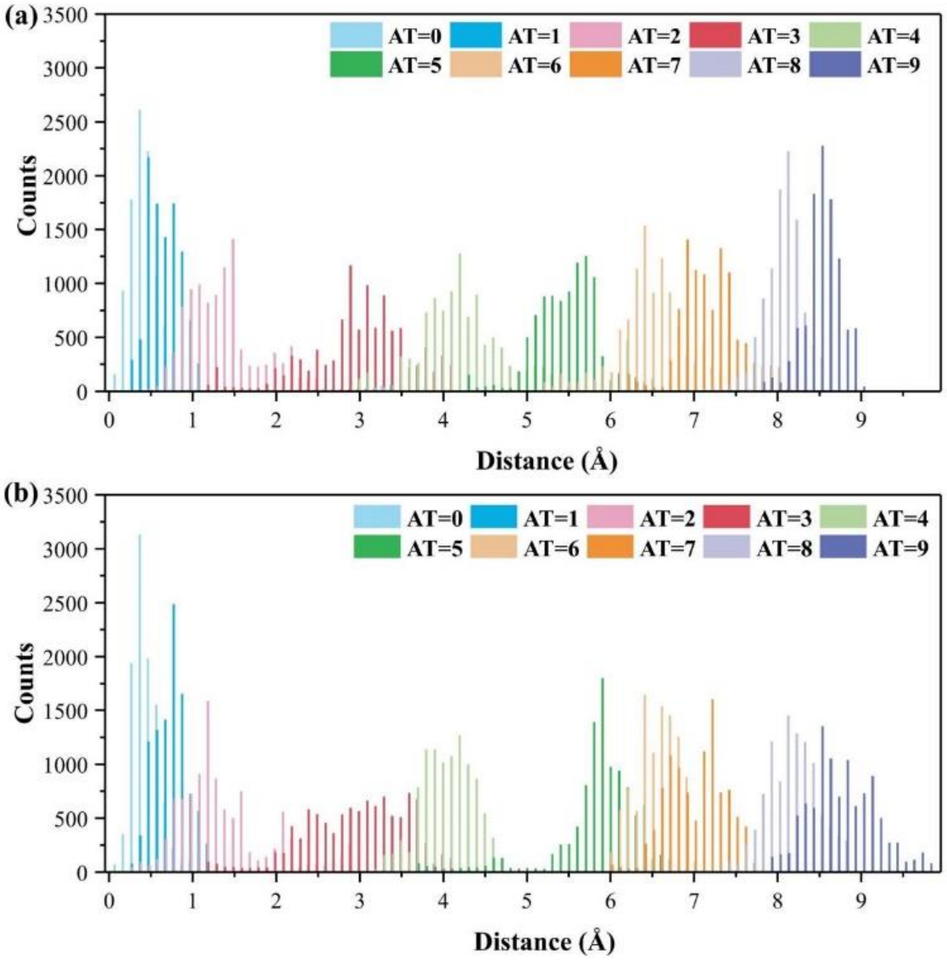

Supplementary Fig. 14. The distribution of distance in every independent umbrella sampling with different AT for acylium ion transport from 8MR channel to 12MR channel. Diffusion in the presence of (a) MeOH and (b) DME in 12MR channel.

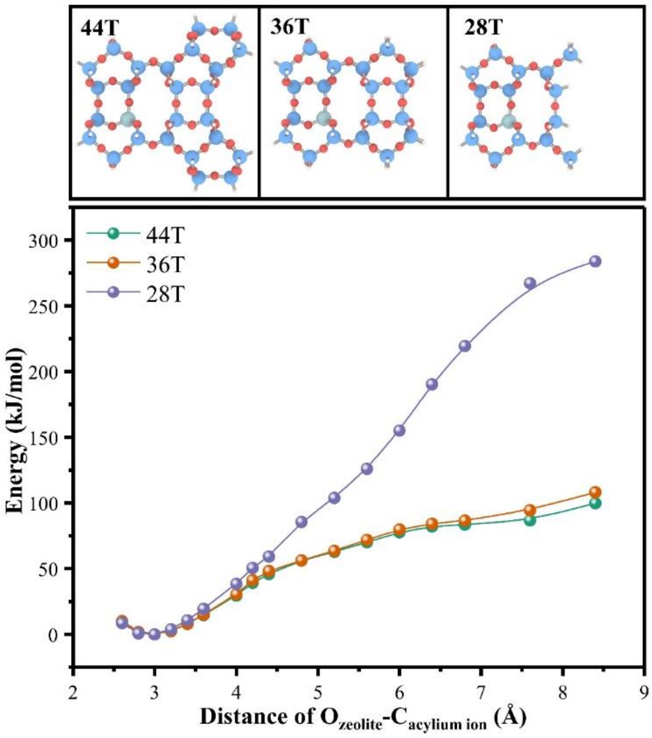

Supplementary Fig. 15. Potential energy surface of acylium ion transporting from 8MR channel to 12MR channel in different size of MOR model. The potential energy surface of 44T model was obtained by the flexible scanning along the increasing C-O distance, and the potential energy surface of other models was directly calculated by single point energy without any optimalization. The method of these potential energy surface was PBE-D3/dgdzvp, and all these results were carried out by Gaussian 09 program.[10]

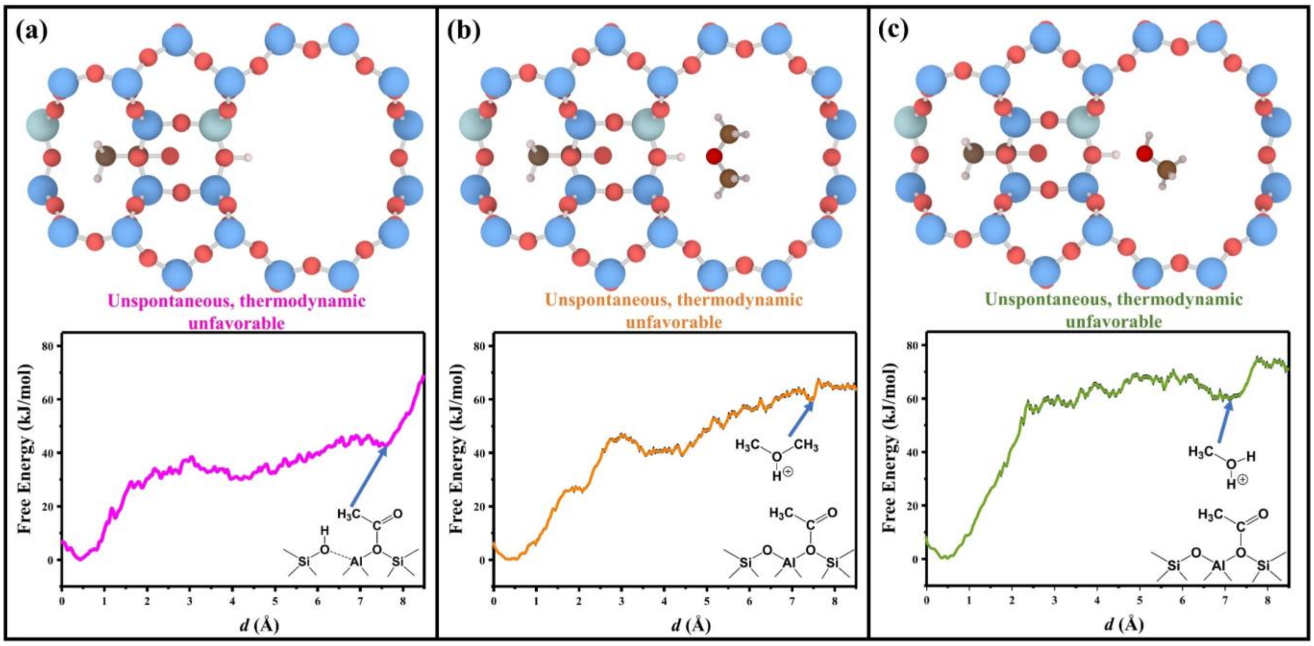

Supplementary Fig. 16. The migration of the acylium ion from the side pocket to the 12MR channel in the presence of additional BAS in 12MR channel. (a) no additional molecule in 12MR channel, (b) one DME in 12MR channel, and (c) one MeOH in 12MR channel.

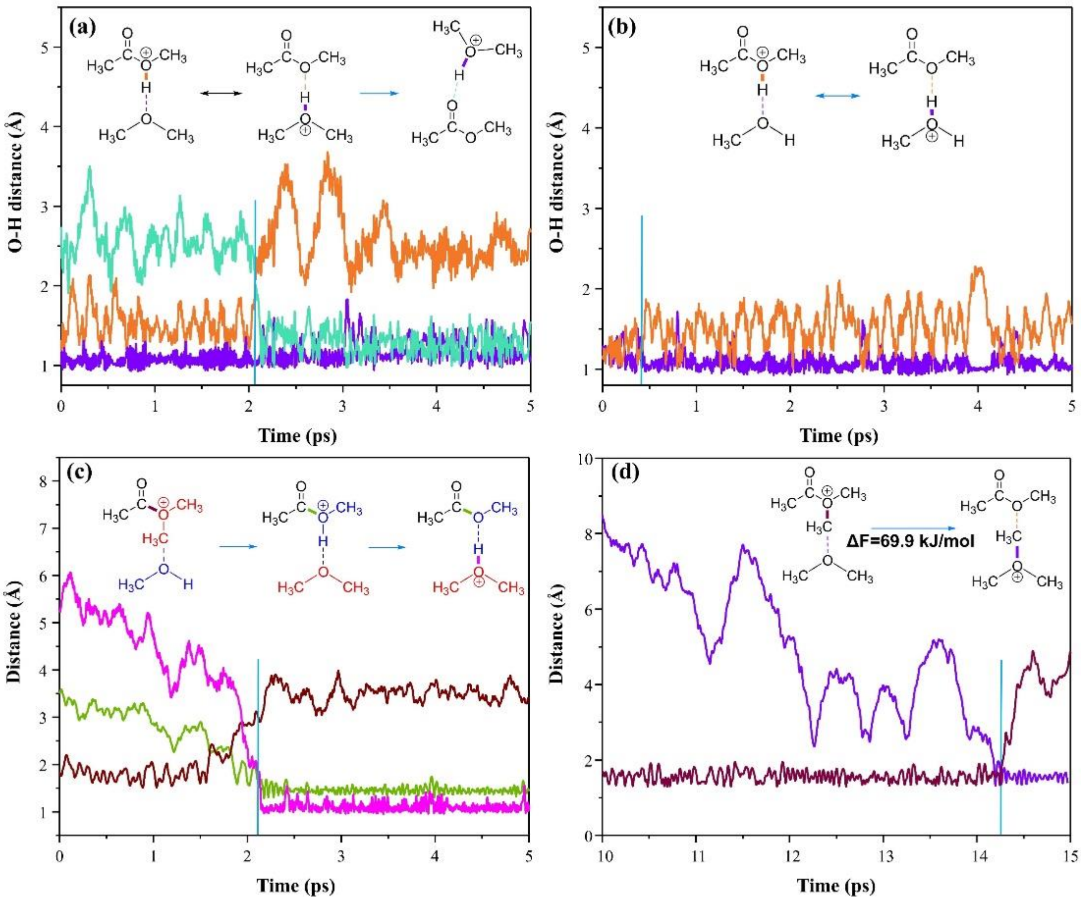

Supplementary Fig. 17. Dynamic evolution of bond distances during Eq. (3) in mordenite. (a) R_1=H, R_2=CH_3, (b) R_1=H, R_2=H, (c) R_1=CH_3, R_2=H, and (d) R_1=CH_3, R_2=CH_3. The color of the curves is consistent with the color of the bonds in the chemical formula.

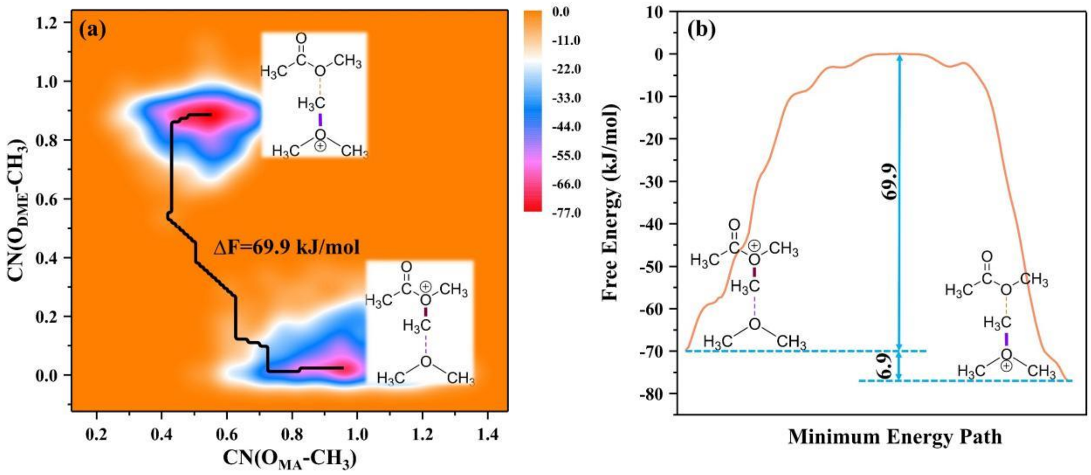

Supplementary Fig. 18. Free energy profile of $MAMe^{+}$ + CH_3OCH_3 → MA + $(CH_3)_3O^{+}$ with the presence of BAS in 8MR channel but occurring in 12MR channel. (a) 2D free energy profile (kJ/mol) with the minimum energy path (black line) and (b) free energy profile of minimum free energy path.

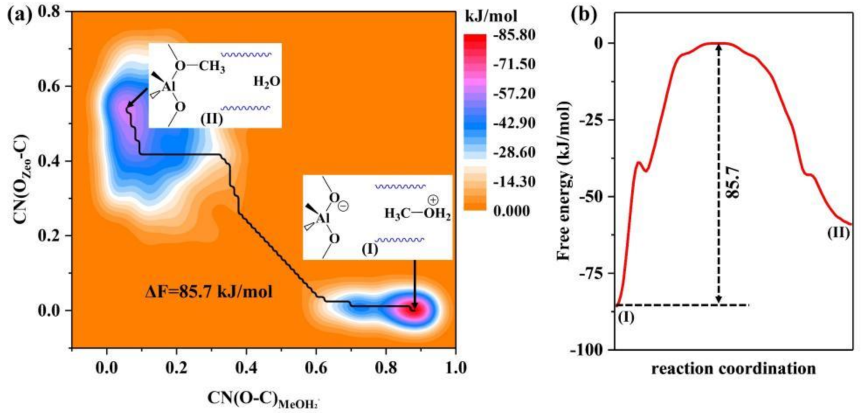

Supplementary Fig. 19. Free energy profile of $MeOH_2^+$ dehydration to SMS in MOR-8MR at 473 K. (a) 2D free energy profile (kJ/mol) with the minimum energy path (black line) and (b) free energy profile of minimum free energy path.

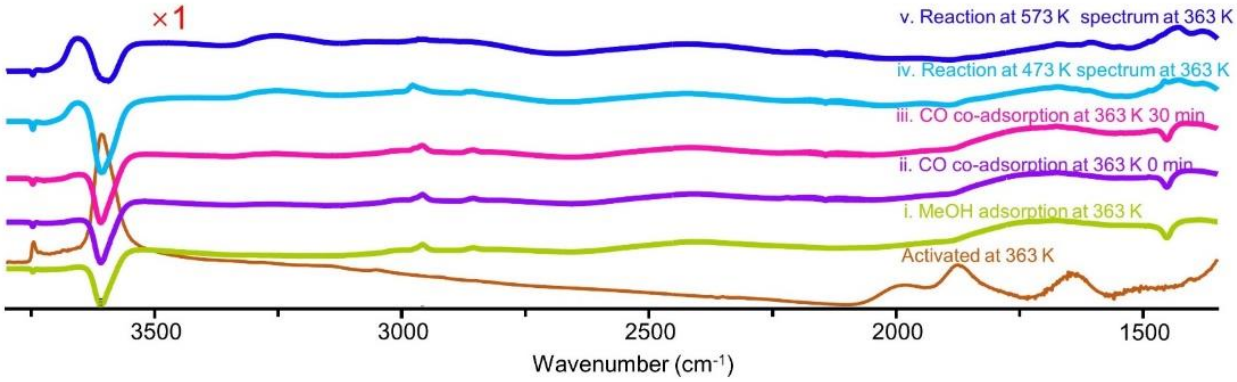

Supplementary Fig. 20. The FTIR spectra collected in whole medium infrared range. Carbonylation substrates adsorbed: (i) MeOH adsorption at 363 K, (ii and iii) MeOH and CO co-adsorption at 363K, as well as the FTIR spectra collected at 363 K after carbonylation reactions at (iv) 473K and (v) 573K.

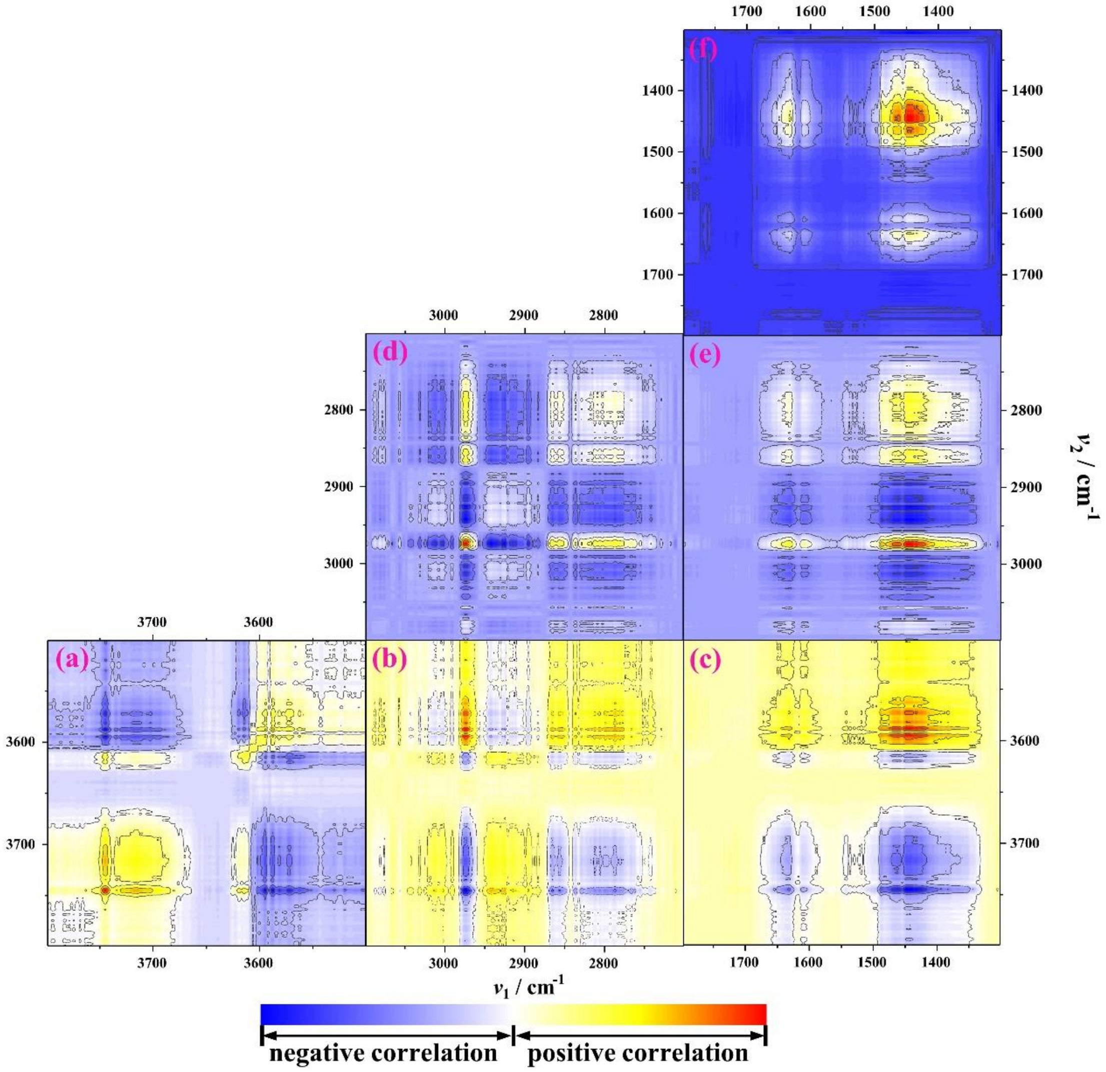

Supplementary Fig. 21. 2-D COS maps of the rapid scan FTIR spectra in the three spectral regions. 3800–3500 cm^{-1}, 3100–2700 cm^{-1}, and 1800–1300 cm^{-1} of O–H stretching, C–H stretching, and C=O stretching together with C-H bending vibrations, respectively. (a) 3800–3500 cm^{-1} × 3800–3500 cm^{-1}, (b) 3800–3500 cm^{-1} × 3100–2700 cm^{-1}, (c) 3800–3500 cm^{-1} × 1800–1300 cm^{-1}, (d) 3100–2700 cm^{-1} × 3100–2700 cm^{-1}, (e) 3100–2700 cm^{-1} × 1800–1300 cm^{-1}, and (f) 1800–1300 cm^{-1} × 1800–1300 cm^{-1}.

Supplementary Table 2. Parameters in MTD for different reactions in this work.

reactants	products	Zeolites	CV1	CV2	W(CV1)	W(CV2)	Hills
Surface acetyl + MeOH	MA + BAS	MOR-12MR	CN(C_{acetyl}-O_{MeOH})	CN(O_{Zeo}-H_{MeOH})	0.035	0.035	3 kJ/mol
Surface acetyl + DME	MA + SMS	MOR-12MR	CN(C_{acetyl}-O_{DME})	CN(O_{Zeo}-C_{DME})	0.035	0.035	3 kJ/mol
Surface acetyl + MeOH	MA + BAS	MOR-8MR	CN(C_{acetyl}-O_{Zeo})-CN(C_{acetyl}-O_{MeOH})	CN(O_{Zeo}-H_{MeOH})	0.035	0.025	3 kJ/mol
$MAMe^+$ + DME	MA + DME	MOR-12MR[a]	CN(C_{MA}-CH_3)	CN(O_{DME}-CH_3)	0.035	0.035	3 kJ/mol
$MeOH_2^+$ + Si-O^--Al	SMS + BAS	MOR-8MR	CN$(C\text{-}O)_{MeOH}$	CN(C_{MeOH}-O_{Zeo})	0.035	0.025	3 kJ/mol
Acylium ion	ketene	MOR-8MR	CN$(C\text{-}H)_{acylium}$	CN(O_{Zeo}-$H_{acylium}$)	0.035	0.035	2 kJ/mol

[a]Reaction occur in MOR-12MR with the Si-O^--Al in MOR-8MR.

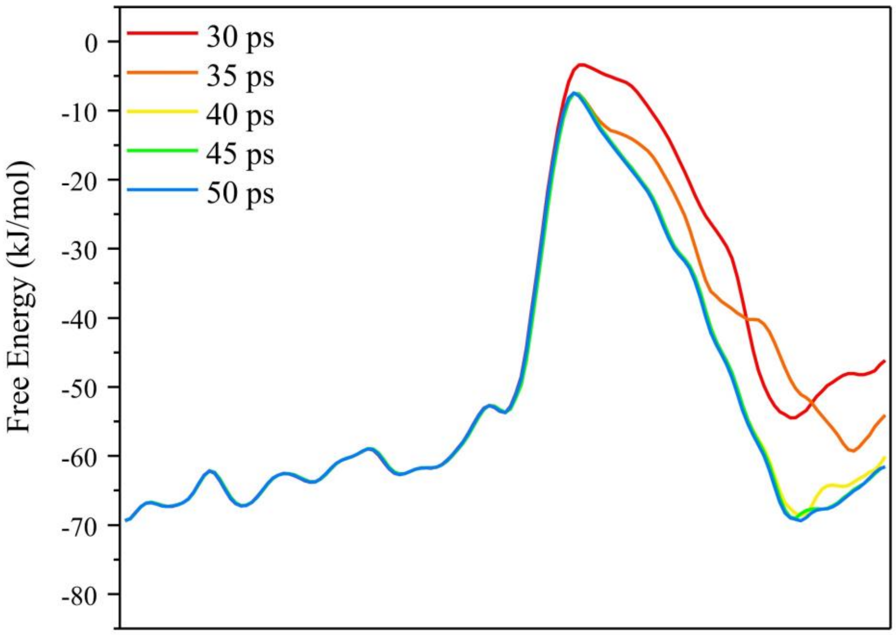

Supplementary Fig. 22. Convergence test of lowest free energy path (LFEP) in the reaction of surface acetyl + MeOH to MA in MOR-8MR. Notably, the free energy barrier is converged when the simulated time is 35 ps according to the same maximum value at the transition state, and LFEP is converged when the simulated time is longer than 45 ps from the perfectly coincident lines between 45 ps and 50 ps.

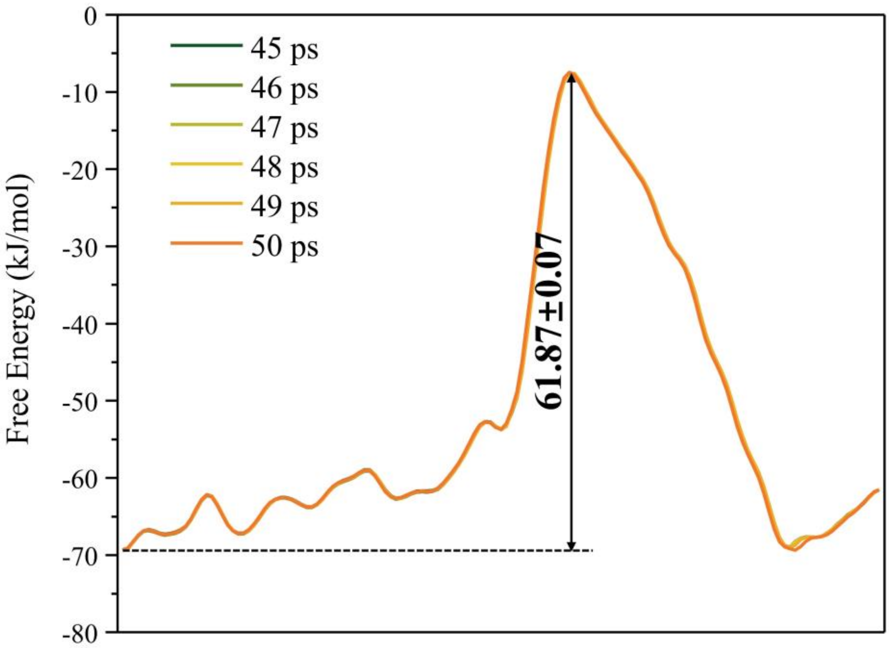

Supplementary Fig. 23. The lowest free energy path (LFEP) in the reaction of surface acetyl + MeOH to MA in MOR-8MR with the different MTD simulated time to estimate the error bar of free energy barrier after reaction equilibrium.

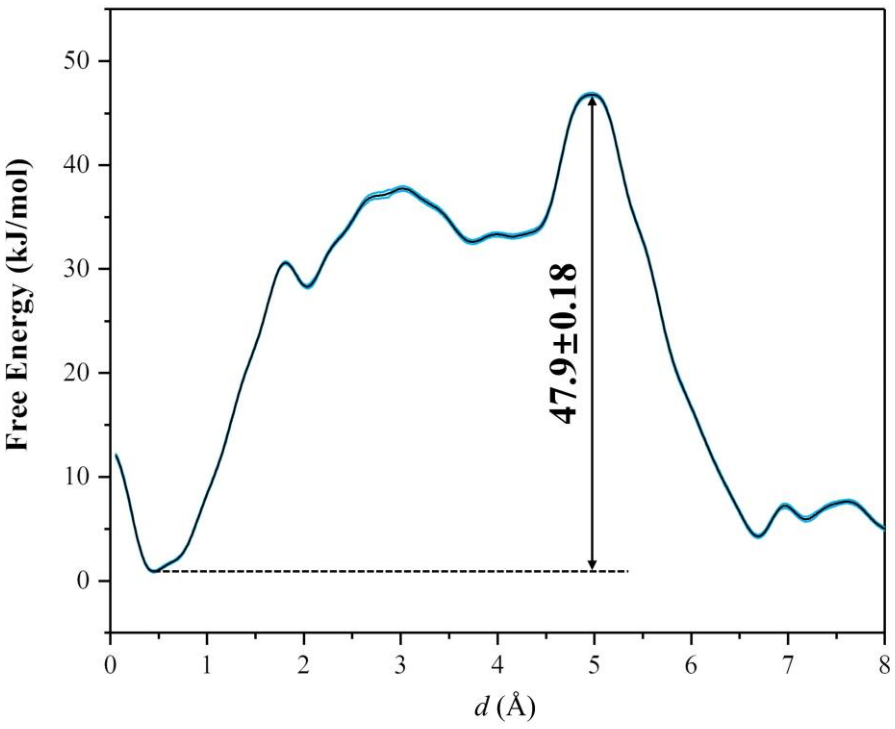

Supplementary Fig. 24. Free energy profile with error bar of the movement of the acylium ion into the 12MR channel previously occupied by DME and the further formation of $MAMe^+$, i.e., the described process in **Fig. 5b**.

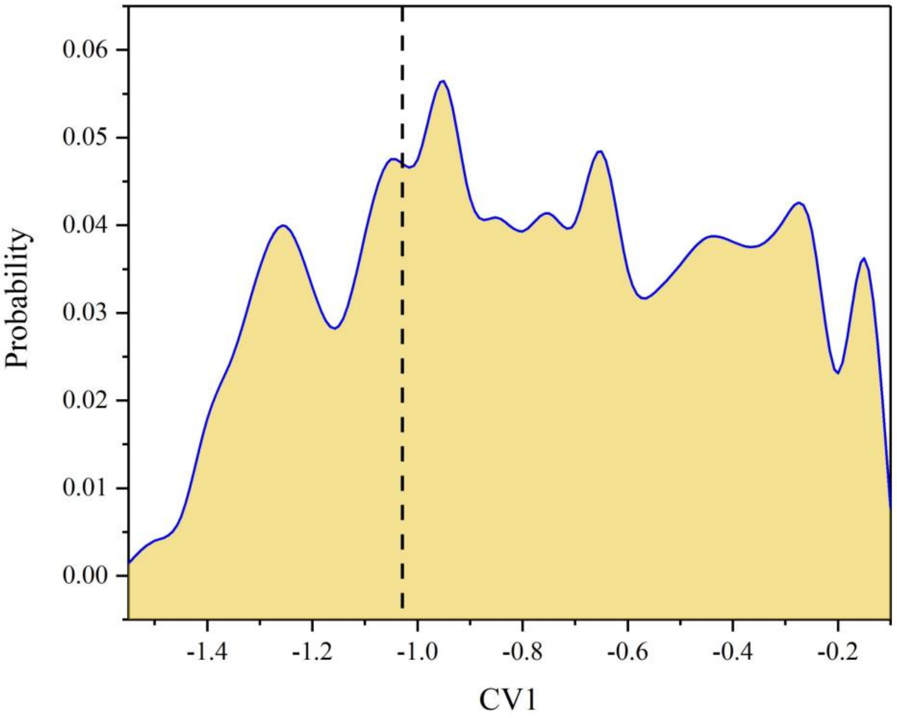

Supplementary Fig. 25. Probability distribution functions of approximation to the CV1 determined using the AIMD-MTD simulations for the reactant configurations (surface acetyl + MeOH in MOR-8MR). Dashed lines indicate the reference reactant states used in the free energy calculations.

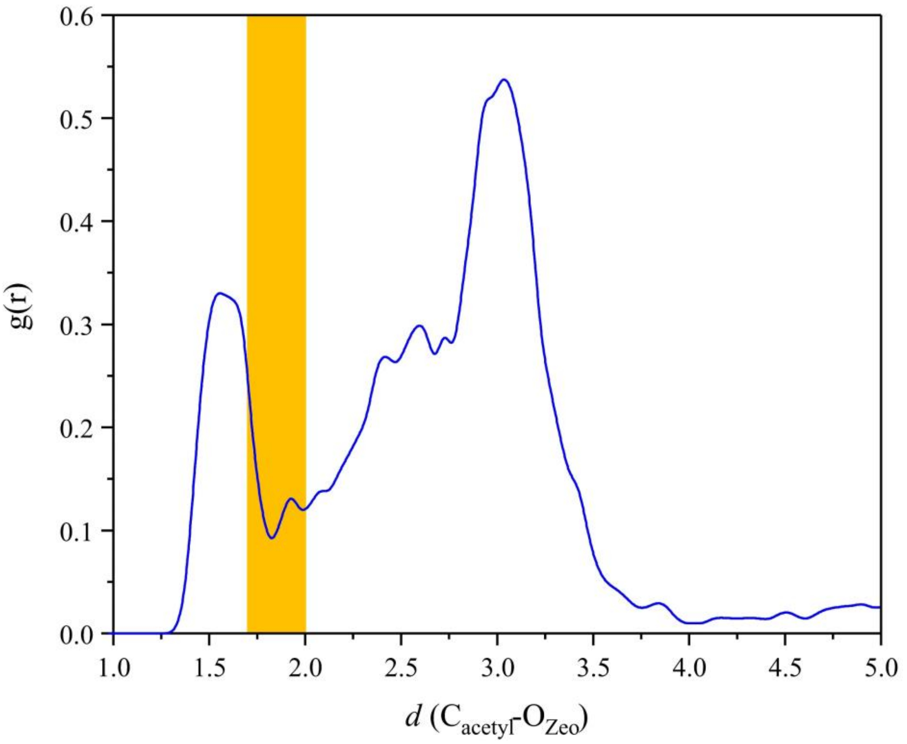

Supplementary Fig. 26. Radial distribution functions (g(r)) for the atoms pair of C_{acetyl} and O_{Zeo} during the AIMD-MTD simulation of surface acetyl + MeOH to MA in MOR-8MR. The highlighted region (1.70 Å ~ 2.00 Å) can be approximately considered as the transition state used in the free energy calculations.

Supplementary References

1 Peters, B. Reaction coordinates and mechanistic hypothesis tests, *Annu. Rev. Phys. Chem.* **67**, 669-690 (2016).

2 Nastase, S. A., Cnudde, P., Vanduyfhuys, L., De Wispelaere, K., Van Speybroeck, V., Catlow, C. R. A. & Logsdail, A. J. Mechanistic insight into the framework methylation of H-ZSM-5 for varying methanol loadings and Si/Al ratios using first-principles molecular dynamics simulations, *ACS Catal*., **10**, 8904-8915 (2020).

3 Souaille, M. & Roux, B. Extension to the weighted histogram analysis method: combining umbrella sampling with free energy calculations, *Comput. Phys. Commun.* **135**, 40-57 (2001).

4 Mitoraj, M. P., Michalak, A. & Ziegler, T. A combined charge and energy decomposition scheme for bond analysis, *J. Chem. Theory. Comput.* **5**, 962-975 (2009).

5 Te Velde, G. T. et al. Chemistry with ADF, *J. Comput. Chem.* **22**, 931-967 (2001).

6 Van Lenthe, E. & Baerends, E. J. Optimized Slater‐type basis sets for the elements 1‐118, *J. Comput. Chem.* **24**, 1142-1156 (2003).

7 Johnson, E. R., Keinan, S., Mori-Sanchez, P., Contreras-Garcia, J., Cohen, A. J. & Yang, W. Revealing noncovalent interactions, *J. Am. Chem. Soc.* 132, 6498-6506 (2010).

8 Lu, T. & Chen, F. Multiwfn: a multifunctional wavefunction analyzer, *J. Comput. Chem*. **33**, 580-592 (2012).

9 Humphrey, W., Dalke, A. & Schulten, K. VMD: visual molecular dynamics, *J. Mol. Graphics* **14**, 33-38 (1996).

10 Frisch, M. et al. Gaussian, Inc., Version B01, Wallingford CT (2009).

11 Wilson, P. J., Bradley, T. J. & Tozer, D. J. Hybrid exchange-correlation functional determined from thermochemical data and ab initio potentials *J. Chem. Phys.* **115**, 9233−9242 (2001).

12 Jensen, F. Basis set convergence of nuclear magnetic shielding constants calculated by density functional methods, *J. Chem. Theory Comput.* **4**, 719−727 (2008).

13 Hoffmann, F., Li, D. W., Sebastiani, D., & Brüschweiler, R. Improved quantum chemical NMR chemical shift prediction of metabolites in aqueous solution toward the validation of unknowns *J. Phys. Chem. A* **121**, 3071−3078 (2017).
