# Supplementary material for: Charge-separation driven mechanism via acylium ion intermediate migration during catalytic carbonylation in mordenite zeolite
Source: Nat Commun. 2022 Nov 19;13:7106. doi: 10.1038/s41467-022-34708-5 (PMC9675746; doi:10.1038/s41467-022-34708-5)
Supplement: Supplementary file 3 — Description of Additional Supplementary Files [file 41467_2022_34708_MOESM3_ESM.pdf]

File name: Supplementary Movie 1

Description: Episode of AIMD simulation for the MA formation process between surface acetyl and methanol in 12MR channel of mordenite

File name: Supplementary Movie 2

Description: Episode of AIMD simulation for the MA formation process between surface acetyl and dimethyl ether in 12MR channel of mordenite

File name: Supplementary Movie 3

Description: Episode of AIMD simulation for the MA formation process between surface acetyl and methanol in 8MR channel of mordenite

File name: Supplementary Movie 4

Description: Episode of AIMD simulation for the MA formation process via migrated acylium ion and methanol in mordenite
